# Total Energy Expenditure in Obese Kuwaiti Primary School Children Assessed by the Doubly-Labeled Water Technique

**DOI:** 10.3390/ijerph13101007

**Published:** 2016-10-13

**Authors:** Lena Davidsson, Jameela Al-Ghanim, Tareq Al-Ati, Nawal Al-Hamad, Anwar Al-Mutairi, Lulwa Al-Olayan, Thomas Preston

**Affiliations:** 1 Food and Nutrition Program, Environment and Life Sciences Research Center, Kuwait Institute for Scientific Research, P.O. Box 24885, Safat 13109, Kuwait; jsager@kisr.edu.kw (J.A.-G.); tareqalaati@gmail.com (T.A.-A.); Anwar.kisr@hotmail.com (A.A.-M.); lolayan@kisr.edu.kw (L.A.-O.); 2 The Public Authority for Food and Nutrition, Bayan 43600, Kuwait; nawal.alhamad@gmail.com; 3 Stable Isotope Biochemistry Laboratory, Scottish Universities Environmental Research Centre, Rankine Avenue, Scottish Enterprise Technology Park, East Kilbride, Glasgow G75 0QF, UK; tom.preston@glasgow.ac.uk

**Keywords:** total energy expenditure, body composition, obesity, children

## Abstract

The aim of this pilot study was to assess body composition and total energy expenditure (TEE) in 35 obese 7–9 years old Kuwaiti children (18 girls and 17 boys). Total body water (TBW) and TEE were assessed by doubly-labeled water technique. TBW was derived from the intercept of the elimination rate of deuterium and TEE from the difference in elimination rates of ^18^O and deuterium. TBW was used to estimate fat-free mass (FFM), using hydration factors for different ages and gender. Fat mass (FM) was calculated as the difference between body weight and FFM. Body weight was not statistically different but TBW was significantly higher (*p* = 0.018) in boys (44.9% ± 3.3%) than girls (42.4% ± 3.0%), while girls had significantly higher estimated FM (45.2 ± 3.9 weight % versus 41.6% ± 4.3%; *p* = 0.014). TEE was significantly higher in boys (2395 ± 349 kcal/day) compared with girls (1978 ± 169 kcal/day); *p* = 0.001. Estimated physical activity level (PAL) was significantly higher in boys; 1.61 ± 0.167 versus 1.51 ± 0.870; *p* = 0.034. Our results provide the first dataset of TEE in 7–9 years old obese Kuwaiti children and highlight important gender differences to be considered during the development of school based interventions targeted to combat childhood obesity.

## 1. Introduction

Obesity and overweight, and related negative health effects, are well-recognized public health problems worldwide, representing one of the major challenges for development in the 21st century [1]. As in many countries in the Gulf region, the lifestyle in Kuwait is characterized by easy access to food, including fast food, and a sedentary lifestyle. The high prevalence of overweight and obesity during childhood is of particular concern due to its long-term effects on the health and well-being of the population. Recent data based on the Kuwait Nutrition Surveillance System [2] reports that 15% of boys and 19% of girls (5 to <10 years) are overweight; the corresponding value for obesity is 20%. These data clearly emphasize the urgent need to develop strategies to combat overweight and obesity, with special focus on children. Globally, childhood obesity is of priority in nutrition and public health and the importance of tackling this public health problem is highlighted by the World Health Organization’s establishment of the high-level Commission on Ending Childhood Obesity [3].

School-based interventions provide an interesting approach to combating childhood obesity. A review by Waters et al. [4], based on a meta-analysis on 37 studies of 27,946 children, reported that interventions were effective in reducing adiposity, although not all individual interventions were effective and there was a high level of heterogeneity. The authors conclude that interventions targeted at school children can be effective, in particular when focused on younger children. In Kuwait, efforts to combat childhood obesity have focused on the development of interventions targeted to primary schoolchildren. As part of a larger initiative, we are currently exploring opportunities to use state-of-the-art stable isotope techniques to monitor and evaluate the efficacy of interventions by assessing changes in body composition, as well as total energy expenditure (TEE). Previous studies most often used conventional techniques, primarily based on anthropometric measures, such as body weight, to evaluate the efficacy of interventions, resulting in modest reductions of body mass index (BMI) z-scores. However, overweight and obesity are defined as abnormal or excessive fat accumulation that may impair health [5] and while BMI is a simple index commonly used to classify overweight and obesity, it does not provide a measure of body fat. For more detailed information about body composition, other methods are clearly needed to assess the different compartments of the body, i.e., fat-free mass and fat mass. Total body water (TBW) assessment by stable isotope dilution is a reference method to estimate fat-free mass [6,7]. Consequently, fat mass can be calculated as the difference between body weight and fat-free mass. It is expected that by using more sophisticated techniques, new data on changes in body composition, in particular reduced body fat, can be demonstrated also in the absence of weight loss. Recent data based on primary school children in Kuwait [8] clearly demonstrated the importance of body composition assessment as children with high percentages of body fat were classified as normal weight and overweight based on BMI-for-age z-scores [9]. These data thus support a discussion by Liu et al. [10] regarding the lack of sensitivity in defining overweight and obesity by the World Health Organization (WHO) criteria.

Changes in physical activity related to school-based interventions are notoriously difficult to demonstrate as activities in school are limited to a relatively small proportion of the day’s activities. The stable isotope technique, using doubly-labeled water (DLW) with analysis of stable isotope ratios in urine collected over several days, offers a measure of TEE without interfering with normal activities. DLW technique is considered the “gold standard” and has been used in nutrition research in humans for many years, for example to validate various techniques to assess energy expenditure in children and adolescents [11], as well as to contribute new data on energy requirements [12,13,14].

The aim of this pilot study was to assess TBW and TEE in a small group of obese primary schoolchildren to provide the first dataset of TEE in 7–9 years old Kuwaiti boys and girls. This study was an integral part of the overall aim of introducing state-of-the-art stable isotope techniques as assessment tools for future lifestyle interventions aimed at combating childhood obesity in Kuwait. The results were expected to provide some insight into differences in TEE between obese boys and girls to be considered for the development of future life style interventions targeted to this age group.

## 2. Materials and Methods

### 2.1. Ethical Standards

The study was conducted according to the guidelines laid down in the Declaration of Helsinki and all procedures were approved by the Ethical Committee of the Ministry of Health, Kuwait (approval number 18186), written informed consent was obtained from all subjects’ parents.

### 2.2. Study Population

Government primary schools were selected for implementation of the study in collaboration with the Ministry of Education, based on previous experience [8]. Body weight and height were measured in all children 7–9 years old available on the days of screening in three schools. In total, 518 girls and 440 boys in grades 2–3 were included in the screening study. All children were Kuwaiti nationals and ethnic Arabs. Body weight (to the nearest 0.1 kg) was measured using a digital scale (SECA, Hamburg, Germany) and height was measured using a portable stadiometer (Holtain, Crymych, UK) to the nearest 1 mm. All measurements were made in triplicate. Mean values were used to calculate BMI and were evaluated against WHO’s growth standards based on BMI-for-age z-scores [9]. A convenience sample of 36 obese children (18 girls and 18 boys) were enrolled into the pilot stable isotope study. Inclusion criteria were: (1) boys and girls 7–9 years old (2) classified as obese based on BMI for age z-score (>+2SD); and (3) parental consent. Exclusion criteria included metabolic diseases, such as diabetes or conditions which could potentially interfere with physical activity; for example, asthma. Medical information was provided by the parents. Parents were informed about the study in writing and a consent form was provided for signature.

### 2.3. Stable Isotope Doses

Individual doses consisting of 4 g of 99% deuterium oxide and 40 g of 10% H_2_^18^O were estimated to be appropriate for a 14 day TEE study in obese Kuwaiti primary school children. The dose preparation was completed in three stages within the same working day. Firstly, a stock dose was prepared gravimetrically in a 5 L glass bottle from supplier’s materials which had been brought to room temperature before opening (10 atom % H_2_^18^O; 99.9 atom % ^2^H_2_O; Cambridge Isotope Laboratories Inc., Andover, MA, USA). The container was mixed thoroughly before doses were aliquotted. Secondly, individual doses were prepared gravimetrically in coded, leak-proof, wide-mouth, polypropylene containers (Nalgene; Nalge Nunc International, Rochester, NY, USA) by transfer from a 100 mL measuring cylinder. All doses were prepared to within 0.3% of the mean weight and stored, sealed and refrigerated, until the day of administration. The third stage of the procedure used the residual stock dose to prepare a “diluted dose” for analysis. The latter was prepared gravimetrically, using local drinking water (Kadhmah water, Kuwait Institute for Scientific Research, Safat, Kuwait), to a dilution representing that expected on the first day of the study. A sample of local drinking water was stored for analysis with the diluted dose to ensure that all unknown samples were analysed in comparison to a known dilution of the study dose [6].

### 2.4. DLW Administration and Urine Collections

All dose administrations were made on a Monday or Tuesday morning to avoid missing any critical data points during weekends. A baseline urine sample was collected and measurements of body weight and height were made before intake of DLW. Spot urine samples were collected in wide-mouthed plastic jugs, labeled with each child’s ID number. Children were instructed to collect urine directly in the containers. Date and time were registered and two cryo-vials were filled with urine, 4–5 mL urine/vial, and placed in Ziploc bags. Vials and Ziploc bags were labeled with the child’s ID number and date and stored frozen (−20 °C) until analysis.

Each child consumed his/her dose directly from the dose bottle, using a drinking straw, under close supervision. The dose bottles were rinsed with 50 mL local drinking water twice (Kadhmah water, Kuwait Institute for Scientific Research, Safat, Kuwait) and the rinsing water was consumed to ensure 100% intake of the dose. Intake of food and drink, as well as vigorous physical activity, was restricted during one hour after intake of the dose. During this time, all children were kept under observation and engaged in sedentary activities. Children returned to class one hour post-dosing. No further restrictions on diet or physical activity were imposed during the study. Urine samples were collected each morning (weekdays) at approximately the same time as baseline samples were collected. On Day 14, the last urine sample was collected and body weight and height were measured.

### 2.5. IRMS Analyses of Urine Samples

Analysis of ^2^H and ^18^O abundance was performed following gas exchange using a Nu Instruments Horizon IRMS (Nu Instruments, Wrexham, UK). Locally-prepared water standards were prepared and analyzed against international standards as described previously [8]. Deuterium abundance of urine samples and standard waters was analyzed by equilibration of H_2_O vapor with H_2_ (10% H_2_, gas balance helium) over a platinum catalyst at 30 °C. Once H_2_ analysis was complete, the IRMS retuned automatically on CO_2_ and established stable operating conditions before undertaking sample gassing (5% CO_2_ in helium gas), equilibration and analysis of ^18^O abundance of waters in all tubes as CO_2_. Urine and standards were analyzed with three measurements of duplicate samples. At the end of each sample batch, all data were saved automatically and imported into an Excel template where the calculation of TBW, TEE and quality control parameters could be performed.

### 2.6. TBW and TEE Calculations

TBW was calculated from stable isotope dilution spaces, based on the intercept of the elimination plot of deuterium and TEE was calculated from the stable isotope elimination rate constants and “pool space” [6]. TBW was used to estimate fat-free mass (FFM), using hydration factors for different ages and gender [15], using the following equation: FFM (kg) = TBW (kg)/hydration coefficient (%). Hydration factors used in this study were 76.8% and 76.2% for boys 7–8 years old and 9–10 years old, respectively. For girls, the corresponding hydration factors are 77.6% and 77.0%. The hydration factors are reproduced with permission from the author in the International Atomic Energy Agency (IAEA) document [7]. Fat mass (FM) was calculated as the difference between body weight and FFM. Percent TBW, FFM, and FM was calculated as (TBW (kg)/body weight (kg) × 100), (FM (kg)/body weight (kg) × 100) and (FFM (kg)/body weight (kg) × 100), respectively.

Schoeller’s equations, as proposed by Goran et al. [16], were used to calculate TEE. TEE, expressed as kcal/day, was calculated based on the complete 14 day protocol, as well as based on the first seven days.

### 2.7. Resting Energy Activity, Physical Activity Level and Activity Energy Expenditure Estimates

Resting energy expenditure (REE) was predicted based on age, sex, and body weight, as described by Schofield [17], and in accordance with the FAO/WHO/UNU Expert Consultation [12]. In this study, we used the equations for children 3–10 years (kcal/day): 22.706 × BW (kg) + 504.3 (boys) and 20.315 × BW (kg) + 485.9 (girls). The equations are included in [12]. Physical activity level (PAL) was estimated based on the ratio TEE/REE. Activity energy expenditure (AEE) was calculated as TEE minus REE. REE and AEE are expressed as kilocalories (kcal) per day.

### 2.8. Statistics

Statistical evaluations were made using SPSS version 22.0 (IBM SPSS Statistics 22, IBM Corporation, Armonk, NY, USA). Quantitative variables were confirmed to be normally distributed using the Shappiro-Wilk test. Results are expressed as mean and SD. Independent student’s *t*-test was used to compare parameters of body composition and energy expenditure between boys and girls. Paired *t*-test was used to compare parameters at baseline versus 14 days, as well as TEE based on seven versus 14 days. Associations between various quantitative variables were tested by Pearson correlation. *p*-Values < 0.05 are referred to as statistically significantly different.

## 3. Results

### 3.1. Study Population

The initial screening study identified 97 (22.0%) of boys and 102 (19.6%) of girls as obese, i.e., had a BMI for age z-score >+2 SD. Among the obese children, 52 boys (53.6%) and 37 girls (36.3%) had a BMI for age z-score >+3 SD. Corresponding values for overweight were 76 (17.2%) and 100 (19.3%) for boys and girls, respectively. Thirty-six children (18 girls and 18 boys) in grades 2–3 were enrolled into the pilot study and 35 completed the TEE study protocol. One boy was excluded from the study before dose administration as he was unable to provide a baseline urine sample. The age range was 7.2 to 9.3 years (girls) and 7.5 to 9.2 years (boys). All children were classified as obese at baseline (BMI for age z-score >+2 SD), 10 boys (58.8%) and four girls (22.2%) had BMI for age z-score >+3 SD, except one boy who had a BMI for age z-score of +2 SD during the screening but a slightly lower body weight at baseline, resulting in a classification as overweight (BMI for age z-score >+1 SD). Nevertheless, we have included him in the dataset.

Body weight, body height, and BMI were not statistically significantly different between boys and girls at baseline: 40.9 ± 7.3 kg body weight (girls) compared with 43.7 ± 8.9 kg body weight (boys; *p* = 0.325) and 131.9 ± 6.1 cm body height (girls) versus 133.8 ± 6.4 cm body height (boys; *p* = 0.395). BMI was 23.4 ± 3.2 (girls) and 24.2 ± 3.3 (boys; *p* = 0.460). On Day 14, girls weighed 41.3 ± 7.3 kg (*p* = 0.003 compared with baseline) and boys weighed 43.8 ± 9.1 kg (*p* = 0.381 compared with baseline). Body height remained unchanged at 131.9 ± 6.1 cm (girls) and 133.9 ± 6.4 cm (boys). Boys’ BMI was not statistically significantly different at Day 14 (24.2 ± 3.4) compared with baseline (*p* = 0.426) while girls had a higher BMI at the end of the study (23.6 ± 3.0) compared with baseline (*p* = 0.01). Information on all individual parameters is presented in the supplementary files.

### 3.2. IRMS Analyses

Each analytical batch contained four tubes each of a “quality control” water standard at natural abundance and a second standard at an enrichment close to that expected the day following the DLW dose. SD of the water standard close to natural abundance was 0.26 ppm ^2^H and 0.22 ppm ^18^O. SD of the enriched waters was 0.16 ppm ^2^H and 0.74 ppm ^18^O (based on all analyses). Values below 1 ppm are regarded as very satisfactory. The enrichment, the difference between the two QC waters, was 0.30 ppm ^2^H excess 1.22 ppm ^18^O excess which is equally satisfactory.

In total, 385 urine samples were collected. Repeat analysis of all urine samples was undertaken to assess analytical reproducibility resulting in two 14 day TEE data sets per child. IRMS analysis revealed that four individual urine samples (<1% of all samples) had most likely been diluted with water during sample collection which made them unusable. Deuterium natural abundance in baseline urine samples averaged 156.7 ppm (SD 0.46 ppm). ^18^O natural abundance averaged 2004.9 ppm (SD 0.77 ppm). These data are very satisfactory and close to the values of Vienna Standard Mean Ocean Water (VSMOW; IAEA, Vienna, Austria), the international reference water.

Results from this study confirm high-quality data, as defined by IAEA [6]. The average internal precision of analysis was 0.79 ppm (deuterium) and 0.78 ppm (^18^O); the mean R^2^ of the isotope elimination regression lines was 0.9982 (deuterium) and 0.9988 (^18^O); the isotope dilution space ratio was 1.032 (SD 0.009) and thus in agreement with an expected range of 1.01–1.07; isotope enrichment on the final day had a mean of 54.2 ppm deuterium excess and 31.9 ppm ^18^O excess (none of the samples collected from 35 subjects fell below 20 ppm deuterium on the 14th day; only one of the samples collected on Day 14 fell below 16 ppm excess ^18^O); the ratio of elimination rate constants was 1.386 (SD 0.043) in accord with an expected range of 1.1–1.7; the re-sampling error was satisfactory, 1.3% for TEE and 1.2% for TBW.

### 3.3. Body Composition

Body weight was not statistically significant between boys and girls but TBW and FFM (kg and %) were significantly higher in boys compared with girls. FM (kg) was not statistically significantly different between boys and girls, but FM % was significantly higher in girls (Table 1). Information on all individual parameters is presented in the supplementary files.

### 3.4. TEE, AEE, and PAL

In Table 2, information on TEE, based on the complete 14 day protocol, is presented together with predicted REE and estimated AEE and PAL. All parameters were significantly higher in boys compared with girls. Information on all individual parameters is presented in the Supplementary Materials.

A comparison between TEE based on the firsts seven days of the pilot study and the complete 14 days protocol showed that TEE in girls did not differ between the two datasets: 1957 ± 182 kcal/day (7 days) compared with 1978 ± 169 kcal/day (14 days; *p* = 0.156), while boys had a significantly higher TEE when measured over 14 days (2395 ± 349 kcal/day) compared with seven days (2336 ± 307 kcal/day; *p* = 0.007). Information on all individual parameters is presented in the supplementary files.

TEE (14 days) was statistically significantly correlated with body weight, body height, and BMI (*p* < 0.005), as well as with TBW (kg) and FFM (kg) (*p* < 0.0001).

Based on the analytical data, the seven day TEE protocol provided very good quality data and a 50% reduction of the study duration is, thus, feasible in this age group. RMS deviation of TBW analysis was 0.083 kg on a mean of 18.26 kg (all children combined), with an excellent CV of just 0.45%, confirming the good quality data generated in this study.

## 4. Discussion

About one in five primary school children in Kuwait is obese, as demonstrated by results from our screening study based on 958 children 7–9 years old. These results are in line with Ministry of Health data, based on the Nutrition Surveillance System [2], and also highlight that a substantial number of children in this age group are overweight. Thus, with an additional 15%–20% of children being overweight, a large proportion of Kuwaiti children are vulnerable to developing poor health later in life. The etiology of childhood obesity, including the trajectory of accumulating excessive body weight among overweight Kuwaiti children, is largely unknown, but assumed to be due to alterations in the regulation of energy balance. To our knowledge, no information is available on total energy expenditure based on DLW technique in obese Kuwaiti children. The focus of this pilot study was to generate the first dataset of TEE in 7–9 years old obese Kuwaiti boys and girls and to provide some insight into gender differences in TEE to be considered for the development of future life style interventions targeted to this age group.

Using the DLW protocol, body composition assessment is based on the intercept method to estimate TBW. Results from the present pilot study demonstrated, as expected based on our previous study [8], that all study children had a high proportion of body fat (mean % FM > 40 in both boys and girls). Additionally, as expected, girls had a significantly higher level of adiposity, expressed as % body fat, and significantly less FFM compared with boys.

Most importantly, gender differences in TEE were also observed as obese boys had significantly higher TEE than girls. Energy expenditure varies primarily as a function of body size and physical activity and TEE was significantly correlated with body weight, height, and BMI in this small study population. We estimated REE as described above, and calculated AEE and PAL, and found that boys were significantly more active than girls. PAL can be used as an indicator of an individual’s activity level and in this report we refer to the classification according to FAO/WHO/UNU [12] as “light”, “moderate”, and “heavy” physical activity levels based on gender and age. Although boys had a significantly higher PAL than girls in this study, it should be noted that PAL for boys (mean 1.61) is at the lower end of the cut offs for “moderate” physical activity for this age group [12] and there was a wide range in PAL for boys (1.39 to 2.08). Two boys had a PAL representing “heavy” physical activity (≥1.90) while PAL for nine boys represented “light” physical activity. Girls had a mean PAL of 1.51 which is higher than the cut offs for “light” physical activity in this age group but not reaching the “moderate” physical activity level (1.60–1.65). None of the girls reached a PAL representing “heavy” physical activity. Three girls’ PAL indicated “light” physical activity.

In this pilot study, obese girls were less physically active than obese boys. However, it is important to emphasize that our study is limited to only 35 children, and as REE was estimated—not measured—results should be interpreted with caution. We cannot estimate the time spent on physical activity during the day, nor the intensity of different activities, but our preliminary observational studies in government primary schools during this study indicate that Kuwaiti girls generally seem less physically active during recess and physical education classes than boys Government primary schools are segregated based on gender in Kuwait. However, most primary school teachers are female, including in boys’ schools, and our preliminary observations indicate that physical activity is more vigorous in boys’ schools where male teachers work. Future studies, combining TEE with methodology designed to capture time spent on different activities, as well as the intensity of different activities in larger groups of children with different BMI for age z-scores, would provide useful information to better understand the activity patterns of Kuwaiti children.

The Institute of Medicine (IOM) [13] recommends that children should spend an average of 60 min in physical activity of moderate intensity daily. However, the dietary reference intakes are provided for the general healthy population and does not provide any recommendations regarding physical activity levels for weight loss. Recently, IOM [18] recommended that all elementary school students should spend an average of 30 min per day in physical education classes to contribute 50% of the recommendation. In addition, opportunities to increase physical activity during the school day, such as during recess, should be explored. In order to reach this recommendation in Kuwait, major efforts would be needed to increase physical activity during the school day, in particular for girls attending government primary schools, for example, by improving the status of physical education classes within the curriculum and encourage active recess in primary schools. Due to the extreme weather conditions in Kuwait, outdoor activities are not an option during a large part of the year and innovative solutions are needed, both within the school system, as well as to encourage activities outside the school day. The present study was implemented during winter (November–December 2014) when the weather in Kuwait is pleasant and compatible with outdoor activities. We have no information on TEE in obese primary school children during the hot summer months.

From a methodological point of view, the DLW technique has several important advantages, as discussed above, and measurements of TEE have been used to increase our understanding of the regulation of energy balance in children [19]. However, as stressed by Goran and Sun [19], it should be noted that, although a fourteen day DLW protocol is considered relatively long-term, it represents a very short time when compared with the time frame for the development of obesity. On the other hand, the short study duration offers an opportunity to estimate total energy intake. During childhood, energy intake should support a healthy body weight, allow for adequate levels of physical activity and provide energy for growth [14]. As discussed by Swinburn et al. [20], mean TEE can be used to reflect mean energy intake as growth would represent a small (negligible) contribution to the energy needs during a typical TEE study protocol. Based on these assumptions, energy intake was 1977 ± 169 kcal/day and 2395 ± 349 kcal/day for girls and boys in the present study. Accurate data on energy intake is difficult to collect, in particular in children and adolescents, and we are not aware of any previous study reporting on energy intake in obese Kuwaiti primary schoolchildren. The use of DLW methodology offers interesting opportunities to estimate energy intake under unrestricted conditions, for example to validate different dietary assessment methods [21,22,23]. Recently, results based on DLW technique demonstrated an association between higher BMI and male sex with greater underreporting of energy intake among adolescents [24].

As we are interested in using the DLW technique as a monitoring and evaluation tool, it is encouraging to note that our data support the application of a shorter study protocol (seven days) in obese primary schoolchildren in Kuwait. A shorter study duration would have practical benefits for the children participating in the study as well as for the study team. Additionally, significant financial savings can be made with a shorter study protocol as the stable isotope dose can be reduced substantially and analytical costs would be lower. However, as we noted a significant difference in TEE for boys based on seven versus 14 days, our data highlights the importance of strict standardization of the study duration to enable comparisons within, as well as between, study populations. The methodological advantages of DLW technique are numerous and of particular interest as an evaluation tool to monitor changes in TEE and body composition during the implementation of life style interventions. The reproducibility of the DLW technique in longitudinal studies was recently demonstrated [25], confirming its usefulness in this application. In Kuwait, development of culturally-acceptable lifestyle interventions targeted to primary school children is a priority in public health. At the time of planning the present study, a large scale nutrition intervention, the “school snack” program, was implemented in government primary schools. This intervention was considered an interesting opportunity to provide information on nutrition and healthy diets, if expanded to include an educational component. However, this program has since been discontinued and, at the present time, there is no intervention in place to provide school meals and, thus, no infrastructure in place to integrate a lifestyle intervention in government primary schools. There is clearly scope for strengthening the physical education activities in primary schools as our preliminary observations indicated low status within the curriculum and, in particular in girls’ schools, the activities seemed to be of low intensity. However, although school-based programs offer unique opportunities for implementation of public health interventions to combat childhood obesity, a more comprehensive national strategy is needed in countries with high prevalence of childhood obesity. In Kuwait, concerted action is urgently needed to change the obesogenic environment children live in, for example, by addressing marketing of unhealthy foods to children [26]. However, as preventing childhood obesity starts during pregnancy [27], we advocate a strong focus on interventions during early life, including strong support to new mothers to increase the rate of exclusive breastfeeding to six months [28].

## 5. Conclusions

Our results, based on a pilot study, highlight important differences in TEE, body composition, and parameters reflecting physical activity between obese 7–9 years old Kuwaiti boys and girls. These findings will be critical for further development of targeted interventions aiming at combating obesity in this age group. Future school-based interventions aiming at encouraging an active lifestyle in this age group could, for example, focus on the introduction of active recess and more vigorous activities during physical education classes to increase physical activity. In particular, special attention is needed to encourage physical activity among girls attending government primary schools.

An important output of this project was to provide evidence that a 50% reduction of the DLW dose and study duration can be used successfully in this age group. The financial savings related to this approach, as well as the increased feasibility of implementing a shorter TEE protocol in primary schools, increase the practical applications of this “gold standard” methodology. Future studies should expand to include larger groups of children to include normal weight, overweight, as well as obese boys and girls, to provide a better understanding of energy expenditure during childhood. Specific factors of importance to the lifestyle in Kuwait; for example, the influence of outdoor temperature on physical activity, would also contribute useful information relevant to the design and implementation of lifestyle interventions in Kuwait.

## Figures and Tables

**Table 1:** Mean values and standard deviation (SD) for body composition parameters. Total body water (TBW), fat-free mass (FFM), and fat mass (FM) are expressed as kilograms (kg) and percent of total body weight (%). Results are based on 17 boys and 18 girls.

	TBW (kg)	TBW (%)	FFM (kg)	FFM (%)	FM (kg)	FM (%)
Girls, mean	17.2	42.4	22.2	54.7	18.7	45.2
SD	2.17	3.0	2.8	3.9	4.9	3.9
Boys, mean	19.4	45.0	25.2	58.4	18.4	41.6
SD	3.07	3.3	4.0	4.3	5.3	4.3
*p* value	0.018	0.025	0.013	0.014	0.867	0.014

**Table 2:** Total energy expenditure (TEE) based on 14 days data collection, predicted resting energy expenditure (REE), estimated activity energy expenditure (AEE) expressed as kilocalories (kcal) per day, and calculated physical activity level (PAL; TEE/REE) for 17 boys and 18 girls.

	TEE 14 Day (kcal/day)	REE (kcal/day)	AEE (kcal/day)	PAL
Girls, mean	1977	1317	661	1.51
SD	169	148	87	0.870
Boys, mean	2395	1496	900	1.61
SD	349	202	238	0.167
*p* value	0.001	0.005	0.001	0.034

## Supplementary Materials

The following are available online at www.mdpi.com/1660-4601/13/10/1007/s1. Table S1: Identification number, gender, age, body weight, body height, body mass index (BMI) and BMI for age z-score at baseline; Table S2: Identification number, gender, age, body weight, body height, body mass index (BMI) and BMI for age z-score at Day 14; Table S3: Identification number, gender, total body water (TBW), fat free mass (FFM) and fat mass (FM); Table S4: Identification number, gender and total energy expenditure (TEE) based on 14 days data collection, resting energy expenditure (REE), activity energy expenditure (AEE) and physical activity level (PAL); Table S5: Identification Number, Gender and Total Energy Expenditure (TEE) Based on the First 7 days and the Complete 14 days Protocol.

## Abbreviations

The following abbreviations are used in this manuscript:

BMI	Body Mass Index
DLW	Doubly Labeled Water
FAO	Food and Agriculture Organization of the United Nations
FM	Fat Mass
FFM	Fat Free Mass
IAEA	International Atomic Energy Agency
IOM	Institute of Medicine
IRMS	Isotope Ratio Mass Spectrometry
PAL	Physical Activity Level
TBW	Total Body Water
TEE	Total Energy Expenditure
UNU	United Nations University.
WHO	World Health Organization
