# Supplementary material for: Total Energy Expenditure in Obese Kuwaiti Primary School Children Assessed by the Doubly-Labeled Water Technique
Source: Int J Environ Res Public Health. 2016 Oct 13;13(10):1007. doi: 10.3390/ijerph13101007 (PMC5086746; doi:10.3390/ijerph13101007)
Supplement: Supplementary file 1 [file ijerph-13-01007-s001.pdf]

Supplementary Materials: Total Energy Expenditure in Obese Kuwaiti Primary School Children Assessed by the Doubly Labeled Water Technique

Lena Davidsson, Jameela Al-Ghanim, Tareq Al-Ati, Nawal Al-Hamad, Anwar Al-Mutairi, Lulwa Al-Olayan and Thomas Preston

Table S1 provides an overview of each child's age, body weight, body height, BMI and BMI for age z-score (WHO 2009) at baseline.

Table S1. Identification number, gender, age, body weight, body height, body mass index (BMI) and BMI for age z-score at baseline.

ID/Gender	Age (Years)	Body Weight (kg)	Body Height (cm)	BMI	BMI for Age z-Score
1/female	7.3	56.2	136.2	30.3	>+3SD
2/female	7.5	32.8	125.5	20.8	>+2SD
3/female	7.5	30.8	123.3	20.2	>+2SD
4/female	8.2	36.4	128.9	21.9	>+2SD
5/female	7.7	35.5	124.4	22.9	>+2SD
6/female	8.0	53.6	139.6	27.5	>+3SD
7/female	8.7	41.4	137.7	21.8	>+2SD
8/female	7.8	37.8	129.1	22.7	>+2SD
9/female	7.6	30.6	119.7	21.4	>+2SD
10/female	8.8	43.2	139.5	22.2	>+2SD
11/female	7.2	52.0	130.4	30.6	>+3SD
12/female	8.4	43.0	127.8	26.3	>+3SD
13/female	9.3	45.8	138.1	24.0	>+2SD
14/female	8.6	41.6	135.7	22.6	>+2SD
15/female	8.7	39.8	135.6	21.7	>+2SD
16/female	8.7	37.8	132.4	21.6	>+2SD
17/female	8.6	37.6	132.7	21.4	>+2SD
18/female	8.4	40.6	138.1	21.3	>+2SD
19/male	7.9	41.2	128.0	25.2	>+3SD
20/male	8.5	52.2	140.1	26.6	>+3SD
21/male	8.8	44.6	139.4	23.0	>+2SD
22/male	8.2	35.6	128.7	21.5	>+2SD
23/male	8.6	38.6	126.3	24.2	>+3SD
24/male	8.5	38.0	128.5	23.0	>+2SD
25/male	8.0	31.6	127.4	19.5	>+1SD
27/male	9.0	52.8	138.4	27.6	>+3SD
28/male	7.8	43.0	134.1	23.9	>+3SD
29/male	7.5	40.2	127.8	24.6	>+3SD
30/male	7.6	39.4	129.4	23.5	>+3SD
31/male	7.6	32.6	126.1	20.5	>+2SD
32/male	7.9	66.8	140.4	33.9	>+3SD
33/male	8.9	45.0	135.6	24.5	>+3SD
34/male	9.2	39.6	135.3	21.6	>+2SD
35/male	8.9	46.6	145.3	22.1	>+2SD
36/male	8.7	54.4	142.9	26.6	>+3SD

Table S2 provides an overview of each child's body weight, body height, BMI and BMI for age z-score at Day 14.

Table S2. Identification number, gender, age, body weight, body height, body mass index (BMI) and BMI for age z-score at Day 14.

ID/Gender	Body Weight (kg)	Body Height (cm)	BMI	BMI for Age z-Score
1/female	56.6	136.0	30.6	>+3SD
2/female	32.4	125.5	20.6	>+2SD
3/female	31.2	123.0	20.6	>+2SD
4/female	37.4	128.8	22.5	>+2SD
5/female	35.6	123.7	23.3	>+2SD
6/female	54.4	139.5	28.0	>+3SD
7/female	41.6	137.5	22.0	>+2SD
8/female	37.6	129.4	22.5	>+2SD
9/female	31.6	119.9	22.0	>+2SD
10/female	43.8	139.5	22.5	>+2SD
11/female	51.4	131.0	30.0	>+3SD
12/female	43.4	128.1	26.4	>+3SD
13/female	46.8	138.3	24.5	>+2SD
14/female	41.6	136.3	22.4	>+2SD
15/female	40.4	135.1	22.1	>+2SD
16/female	38.4	132.2	22.0	>+2SD
17/female	38.8	132.8	22.0	>+2SD
18/female	40.8	138.2	21.4	>+2SD
19/male	41.2	128.1	25.1	>+3SD
20/male	53.0	140.4	26.9	>+3SD
21/male	44.6	139.5	22.9	>+2SD
22/male	35.4	128.8	21.3	>+2SD
23/male	38.6	126.3	24.2	>+3SD
24/male	38.6	128.6	23.3	>+2SD
25/male	32.0	127.7	19.6	>+1SD
27/male	53.2	138.5	27.7	>+3SD
28/male	42.8	134.4	23.7	>+3SD
29/male	39.8	128.0	24.3	>+3SD
30/male	39.0	129.4	23.3	>+3SD
31/male	32.2	126.4	20.1	>+2SD
32/male	67.4	140.7	34.0	>+3SD
33/male	44.4	135.9	24.0	>+3SD
34/male	39.6	135.9	21.5	>+2SD
35/male	47.0	145.2	22.3	>+2SD
36/male	55.0	143.4	26.8	>+3SD

Table S3 provides information on body composition (TBW, FFM and FM) expressed as kg and % of total body weight.

Table S3. Identification number, gender, total body water (TBW), fat free mass (FFM) and fat mass (FM).

ID/Gender	TBW (kg)	TBW (%)	FFM (kg)	FFM (%)	FM (kg)	FM (%)
1/female	19.9	35.4	25.7	45.7	30.5	54.3
2/female	14.3	43.6	18.4	56.2	14.4	43.8
3/female	13.4	43.6	17.3	56.2	13.5	43.8
4/female	15.8	43.4	20.4	55.9	16.0	44.1
5/female	15.0	42.3	19.4	54.6	16.1	45.4
6/female	20.2	37.7	26.0	48.7	27.5	51.3
7/female	17.2	41.4	22.2	53.6	19.2	46.4
8/female	16.2	42.9	20.9	55.3	16.9	44.7

9/female	13.7	44.9	17.7	57.9	12.9	42.1
10/female	19.8	45.8	25.6	59.3	17.6	40.7
11/female	20.2	38.8	25.9	49.8	26.1	50.2
12/female	17.0	39.5	22.0	51.1	21.0	48.9
13/female	18.3	39.9	23.6	51.6	22.2	48.4
14/female	18.2	43.8	23.6	56.7	18.0	43.3
15/female	18.4	46.3	23.8	59.9	16.0	40.1
16/female	16.7	44.3	21.7	57.3	16.1	42.7
17/female	16.6	44.2	21.5	57.2	16.1	42.8
18/female	18.3	45.0	23.6	58.2	17.0	41.8
19/male	18.0	43.7	23.5	56.9	17.7	43.1
20/male	21.6	41.3	27.9	53.4	24.3	46.6
21/male	19.9	44.6	25.7	57.6	18.9	42.4
22/male	16.3	45.7	21.2	59.6	14.4	40.4
23/male	16.8	43.4	21.9	56.8	16.7	43.2
24/male	17.7	46.6	23.2	60.9	14.8	39.1
25/male	15.4	48.7	20.1	63.5	11.5	36.5
27/male	20.2	38.4	26.2	49.6	26.6	50.4
28/male	20.1	46.7	25.9	60.2	17.1	39.8
29/male	18.1	45.0	23.6	58.6	16.6	41.4
30/male	20.5	51.9	26.7	67.7	12.7	32.3
31/male	15.9	48.8	20.5	63.0	12.1	37.0
32/male	27.6	41.3	36.0	53.8	30.8	46.2
33/male	19.0	42.2	24.6	54.7	20.4	45.3
34/male	17.8	44.9	23.3	58.7	16.3	41.3
35/male	22.0	47.1	28.4	61.0	18.2	39.0
36/male	23.3	42.8	30.5	56.0	23.9	44.0

In Table S4, information on TEE, based on the complete 14 days data collection protocol, is presented together with predicted REE and estimated AEE and PAL for all study children.

Table S4. Identification number, gender and total energy expenditure (TEE) based on 14 days data collection, resting energy expenditure (REE), activity energy expenditure (AEE) and physical activity level (PAL).

ID/Gender	TEE 14 Days (kcal/day)	REE (kcal/day)	AEE (kcal/day)	PAL
1/female	2271	1628	644	1.40
2/female	1651	1152	499	1.43
3/female	1772	1112	661	1.59
4/female	1917	1225	692	1.56
5/female	1968	1206	761	1.63
6/female	2315	1575	740	1.47
7/female	1868	1327	541	1.41
8/female	1988	1254	734	1.59
9/female	1781	1108	673	1.61
10/female	1960	1364	597	1.44
11/female	2101	1542	559	1.36
12/female	2004	1359	645	1.47
13/female	2164	1416	747	1.53
14/female	1844	1331	513	1.39
15/female	1995	1294	700	1.54
16/female	1963	1254	709	1.57
17/female	1941	1250	691	1.55

18/female	2096	1311	785	1.60
19/male	2209	1440	770	1.53
20/male	2732	1690	1043	1.62
21/male	2299	1517	782	1.52
22/male	1978	1313	666	1.51
23/male	2071	1381	690	1.50
24/male	2275	1367	907	1.66
25/male	1898	1222	676	1.55
27/male	2569	1703	866	1.51
28/male	2395	1481	915	1.62
29/male	2944	1417	1527	2.08
30/male	2660	1399	1262	1.90
31/male	2158	1245	913	1.73
32/male	3126	2021	1105	1.55
33/male	2335	1526	809	1.53
34/male	1950	1403	546	1.39
35/male	2533	1562	970	1.62
36/male	2588	1739	849	1.49

A comparison between TEE based on the first seven days of the study versus the complete 14 day protocol is presented in Table S5.

Table S5. Identification number, gender and total energy expenditure (TEE) based on the first 7 days and the complete 14 days protocol.

ID/Gender	TEE First 7 Days (kcal/day)	TEE 14 Days (kcal/day)
1/female	2240	2271
2/female	1619	1651
3/female	1739	1772
4/female	1842	1917
5/female	1882	1968
6/female	2202	2315
7/female	1781	1868
8/female	1932	1988
9/female	1743	1781
10/female	1926	1960
11/female	2182	2101
12/female	2018	2004
13/female	2237	2164
14/female	1867	1844
15/female	1981	1995
16/female	2062	1963
17/female	1900	1941
18/female	2074	2096
19/male	2180	2209
20/male	2686	2732
21/male	2175	2299
22/male	2025	1978
23/male	2004	2071
24/male	2209	2275
25/male	1857	1898
27/male	2453	2569
28/male	2378	2395

29/male	2660	2944
30/male	2603	2660
31/male	2098	2158
32/male	2989	3126
33/male	2373	2335
34/male	1972	1950
35/male	2545	2533
36/male	2503	2588

© 2016 by the authors; licensee MDPI, Basel, Switzerland. This article is an open access article distributed under the terms and conditions of the Creative Commons by Attribution (CC-BY) license (http://creativecommons.org/licenses/by/4.0/).
